# Impact of neuromuscular block on myocardial injury after non-cardiac surgery (MINS) incidence in the early postoperative stage of older patients undergoing laparoscopic colorectal cancer resection: a randomized controlled study

**DOI:** 10.1186/s12877-024-05125-8

**Published:** 2024-06-12

**Authors:** Yi An, Tianlong Wang, Lixia Li, Zhongjia Li, Chuanyu Liang, Pei Wang, Xuefei Jia, Hongyi Song, Lei Zhao

**Affiliations:** Department of Anesthesiology, Xuanwu Hospital, Capital Medical University, 45 Changchun Street, Xicheng District, Beijing, 100053 China

**Keywords:** MINS, Older patients, Deep neuromuscular block, Moderate neuromuscular block, Sugammadex sodium

## Abstract

**Background:**

Myocardial injury after non-cardiac surgery (MINS) is a common and serious complication in older patients. This study investigates the impact of neuromuscular block on the MINS incidence and other cardiovascular complications in the early postoperative stage of older patients undergoing laparoscopic colorectal cancer resection.

**Methods:**

70 older patients who underwent laparoscopic colorectal cancer resection were separated into the deep neuromuscular block group and moderate neuromuscular block group for 35 cases in each group (n = 1:1). The deep neuromuscular block group maintained train of four (TOF) = 0, post-tetanic count (PTC) 1–2, and the moderate neuromuscular block group maintained TOF = 1–2 during the operation. Sugammadex sodium was used at 2 mg/kg or 4 mg/kg for muscle relaxation antagonism at the end of surgery. The MINS incidence was the primary outcome and compared with Fisher's exact test. About the secondary outcomes, the postoperative pain was analyzed with Man-Whitney U test, the postoperative nausea and vomiting (PONV) and the incidence of cardiovascular complications were analyzed with Chi-square test, intraoperative mean artery pressure (MAP) and cardiac output (CO) ratio to baseline, length of stay and dosage of anesthetics were compared by two independent samples t-test.

**Results:**

MINS was not observed in both groups. The highest incidence of postoperative cardiovascular complications was lower limbs deep vein thrombosis (14.3% in deep neuromuscular block group and 8.6% in moderate neuromuscular group). The numeric rating scale (NRS) score in the deep neuromuscular block group was lower than the moderate neuromuscular block group 72 h after surgery (0(1,2) vs 0(1,2), *P* = 0.018). The operation time in the deep neuromuscular block group was longer (356.7(107.6) vs 294.8 (80.0), min, *P* = 0.008), the dosage of propofol and remifentanil was less (3.4 (0.7) vs 3.8 (1.0), mg·kg^−1^·h^−1^, *P* = 0.043; 0.2 (0.06) vs 0.3 (0.07), μg·kg^−1^·min^−1^, *P *< 0.001), and the length of hospital stay was shorter than the moderate neuromuscular block group (18.4 (4.9) vs 22.0 (8.3), day, *P* = 0.028). The differences of other outcomes were not statistically significant.

**Conclusions:**

Maintaining different degrees of the neuromuscular block under TOF guidance did not change the MINS incidence within 7 days after surgery in older patients who underwent laparoscopic colorectal cancer resection.

**Trial registration:**

The present study was registered in the Chinese Clinical Trial Registry (10/02/2021, ChiCTR2100043323).

## Background

With the number of older adults increases worldwide, the need for surgery among older patients is growing continuously. Physiological characteristics of older patients and perioperative stress response may seriously affect the prognosis. Postoperative cardiovascular adverse events are common perioperative complications in older patients, and Kheterpal et al. confirmed that ≥ 68 years old is an independent risk factor for perioperative cardiovascular adverse events [1].

Myocardial injury after non-cardiac surgery (MINS) has received increasing attention recently. MINS is a myocardial injury with prognostic value caused by myocardial ischemia, which usually occurs within 30 days after non-cardiac surgery [2], and the diagnostic criteria are postoperative elevation of cardiac troponin I (cTnI) or cardiac troponin T (cTnT) [3]. MINS incidence is 16–20% in high-risk patients undergoing non-cardiac surgery [4, 5]. MINS was one of the leading cause of death in patients within 30 days after surgery (15.6%) and the first rank cause of death in patients at 1 year after surgery (6.4%) [6]. It is difficult to identify MINS as more than 90% of MINS have no typical clinical symptoms of myocardial injury or myocardial infarction; however, the mortality rate of patients with occult symptoms is slightly lower than patients with clinical symptoms within 30 days after surgery [7]. A cohort study by Judith et al. on 3224 older patients who had undergone major non-cardiac surgery showed that up to 22% developed MINS within 1–3 days after surgery, which was associated with all-cause mortality one year after surgery [8]. Therefore, early identification and prevention of MINS have great clinical significance in improving the prognosis of older patients.

Abdominal surgery is common in older patients. Previous studies have shown that older patients are prone to postoperative cardiovascular complications due to the stress response caused by abdominal surgery, with a significantly extended length of hospital stay and increased reoperation [9, 10]. Laparoscopic surgery conforms to enhanced recovery after surgery (ERAS) and is currently widely used [11]. However, laparoscopic surgery requires establishing an artificial pneumoperitoneum, during which the increased abdominal pressure compresses the visceral vessels, resulting in decreased cardiac return blood volume and increased peripheral vascular resistance, reducing coronary blood flow and cardiac output (CO), and thus the oxygen supply of organs [12]. These factors increase the risk of myocardial oxygen supply and demand imbalance. The degree of neuromuscular block is essential to intraoperative abdominal cavity pressure. Deep neuromuscular block or moderate neuromuscular block are currently considered suitable for muscle relaxation maintenance in laparoscopic surgery. Combining deep muscle release and low pneumoperitoneum pressure (8–10 mmHg) could lead to better operating conditions for surgeons [13, 14]. However, until now, no studies have reported the effects of different degrees of the neuromuscular block on postoperative MINS incidence and cardiovascular adverse events.

The main objective of this study was to compare the effects of deep and moderate neuromuscular blocks on the incidence of early postoperative cardiovascular adverse events in older patients undergoing laparoscopic colorectal cancer resection. A prospective randomized controlled study was designed and the primary outcome is the MINS incidence during the first 7 days after surgery.

## Methods

This study was approved by the Ethics Committee of Xuanwu Hospital ([2021] 030, March 6, 2021) and registered in the Chinese Clinical Trial Registry (10/02/2021, 2021, ChiCTR2100043323).

This study was conducted from March 2021 to May 2022. Inclusion criteria were older patients of 65–79 years who underwent elective laparoscopic colorectal cancer resection, operation time beyond 2 h, BMI 18.5–35 kg/m^2^, and ASA grade I-III. Exclusion criteria were abnormal preoperative myocardial biomarkers, severe pulmonary complications, liver function Child–Pugh grade B or C, creatinine clearance < 30 ml/min, known or suspected neuromuscular disease, and allergic to the drugs used in this study.

The study process was initiated after written informed consent was obtained from patients. All participants were allocated to deep or moderate neuromuscular groups (1:1, n = 35). Complete randomization was performed with random number table and the allocation concealment was carried out by sequentially numbered opaque sealed envelopes. Blood pressure, electrocardiogram (ECG), pulse oxygen saturation, and Bispectral index (BIS) were monitored, and a 16G peripheral venous access was established. Radial artery catheterization was performed on the side of higher non-invasive blood pressure under local anesthesia with 1%lidocaine. Mean artery pressure (MAP), CO and other hemodynamic indicators were continuously monitored at zero point from the midaxillary line. The degree of neuromuscular block was monitored intraoperatively by the train of four (TOF) and post-tetanic count (PTC). The arm of the patient was extended, and two stimulation electrodes were pasted on the ulnar side of the forearm near the wrist joint with a spacing of 2 cm. The stimulation target was the adductor hallucis muscle with a current of 50 mA and a frequency of 2 Hz. The temperature sensor was attached to the palm, and the acceleration sensor was attached to the thumb.

During anesthesia induction, all patients were given propofol 1 mg/kg, TOF calibration was performed after BIS < 80, and sufentanil 0.3ug/kg and rocuronium 0.6 mg/kg were injected successively. The dosage of rocuronium was calculated according to the standard body weight (kg). Endotracheal intubation was performed after TOF = 0, the tidal volume was 6–8 mL/kg, and the ventilation mode was pressure-controlled volume guaranteed ventilation (PCV-VG). The inhaled oxygen concentration was adjusted to 50%; oxygen flow was 3L/min, I:E = 1:2, respiratory rate 12–18 times per minute and 5–10 cm H_2_O positive end-expiratory pressure (PEEP) to maintain end-expiratory carbon dioxide to 35–45 mmHg. A warm blanket and air blower were used to keep the nasopharyngeal temperature above 36 ℃.

Anesthesia was maintained with total intravenous anesthesia (TIVA). Propofol and remifentanil were used to maintain the BIS 40–60, norepinephrine was pumped continuously with 0.03–0.1 µg/kg/min, and IBP was maintained at ± 20% of the baseline. Blood gas analysis was performed to adjust the acid–base balance. Rocuronium was used continuously until the end of the operation, and the TOF was checked every 5–10 min. In the deep neuromuscular block group, pneumoperitoneum pressure was maintained at 8–10 mmHg with TOF = 0 and PTC 1–2. The moderate neuromuscular block group maintained pneumoperitoneum pressure at 12–14 mmHg with TOF = 1–2. If the surgical area was not exposed satisfactorily, pneumoperitoneum pressure increased at a gradient of 2 mmHg as required by the surgeon, with a maximum of 20 mmHg.

After closing the incision, the surgeons used 10 ml of 0.5% ropivacaine for local anesthesia, and 4 mg ondansetron was administered at the end of the operation. Sugammadex sodium was used at 4 mg/kg for the deep neuromuscular block group or 2 mg/kg for the moderate neuromuscular block group. Extubation was performed until the TOF ratio was > 90% and the patient's consciousness recovered. The postoperative analgesia of the patient was combined with sufentanil 1.5 μg × kg body weight (kg) and normal saline to 100 ml, and the postoperative analgesia was maintained until 48 h after surgery.

The primary outcome of this study was MINS incidence within 7 days after surgery, and cTnI > 0.06 ng/L was clinically significant [15]. The anesthesiologist collected the venous blood of patients before anesthesia induction and 1 day, 2 days, 3 days and 7 days after surgery to test the serum levels of cTnI, CK-MB, Myo and NT-proBNP. The secondary outcomes were the dosage of anesthetics, Leiden-surgical rating scale (L-SRS), length of stay, pain intensity, postoperative nausea and vomiting (PONV), incidence of cardiovascular complications and MAP and CO ratio to the baseline during the operation. Drug dosage was recorded and surgeons used the L-SRS to evaluate surgical area exposure on a scale of 1–5; a score of 1 indicates extremely poor surgical conditions, and a score of 5 indicates optimal surgical conditions. The anesthesiologists recorded the baseline and the changes in MAP and CO during the operation and recorded the times of artificial pneumoperitoneum adjustment. Postoperative follow-up was performed by anesthesiologists blinded to the group.. Numerical rating scale (NRS) scores and PONV of patients at 24 h, 48 h and 72 h after surgery were counted, as well as the incidence of cardiovascular complications, such as arrhythmia, angina pectoris, myocardial infarction and heart failure, were dealt with after surgery.

### Statistical analysis

This study used MINS incidence for sample size estimation, as described in previous literature. The incidence of postoperative MINS in older patients (aged ≥ 60 years) was 19.4%-52.9% according to previous studies when cTnI was used as a biomarker [15, 16]. Accordingly, we estimate that the MINS proportion in the deep neuromuscular block group will be 19.4% on account of the expected slighter impact on circulatory function, while the proportion in the moderate neuromuscular block group is estimated to be 52.9%. Using a power of 80% and α of 0.05 to carry out the two-side test, the calculated sample size is 58. Considering the 20% loss of follow-up, 35 patients were included in each group (n = 1:1).

Statistical data were analyzed by SPSS, and *P* < 0.05 was considered statistically significant. The S-W test was used to analyze normality. MINS incidence was compared with Fisher's exact test. Two independent samples t-test was used to analyze the dosage of anesthetics, length of stay, and MAP and CO ratio to the baseline during the operation. L-SRS, incidence of PONV and cardiovascular complications were analyzed with the Chi-square test and NRS was tested using the Man-Whitney U test.

## Results

**Fig. 1:**
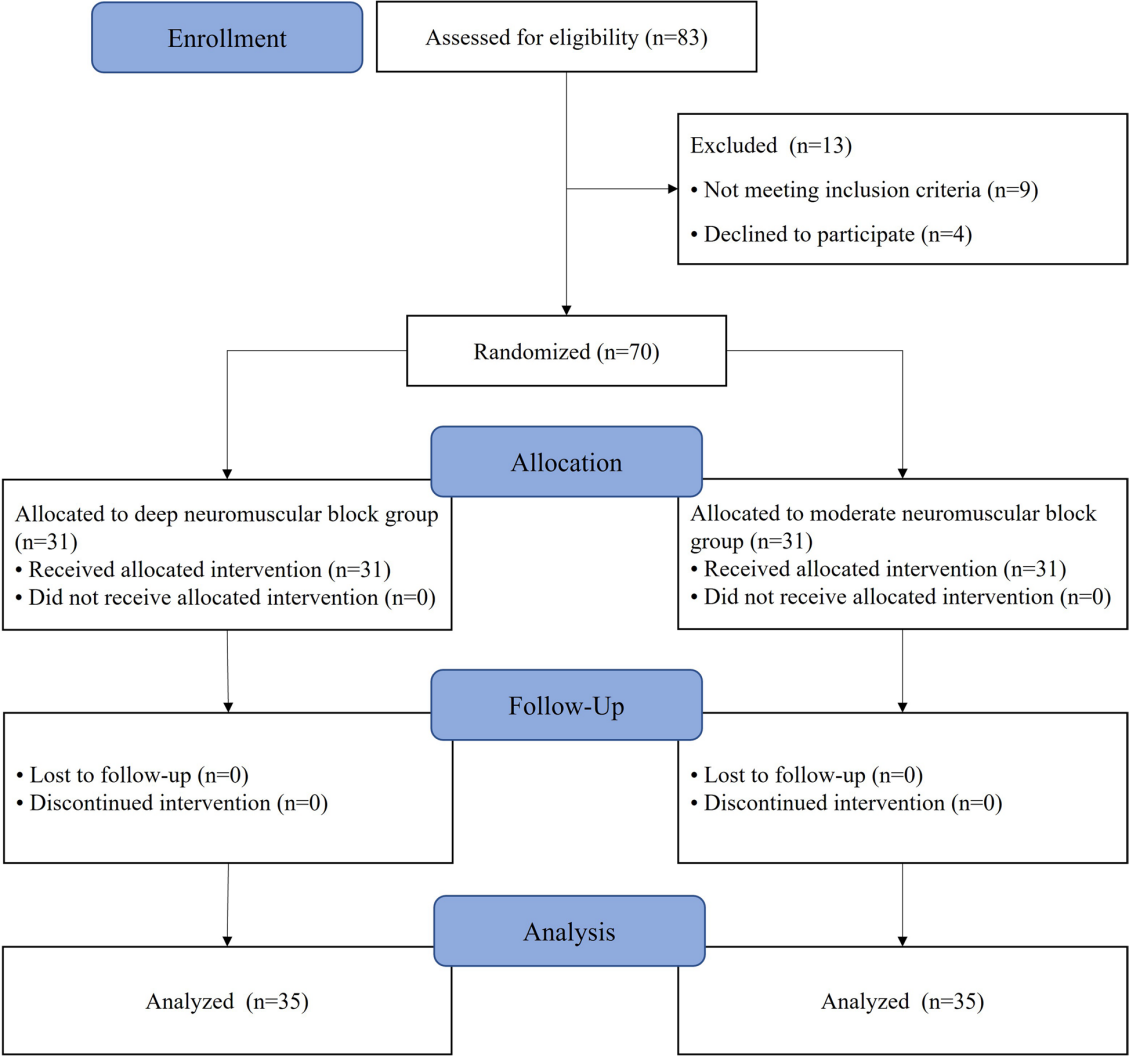
Consolidated Standards of Reporting Trials (CONSORT) flow diagram of trial participants

The participant recruitment was conducted from March 2021 to May 2022. Figure 1 shows the flow diagram based on the CONSORT statement. After assessing 83 participants and excluding 13 patients, 70 patients were eligible and allocated into the deep and moderate neuromuscular groups (1:1, n = 35). The participants completed postoperative follow-up and were included in the final data analysis.

Table 1 shows the baseline data and intraoperative characteristics of the two groups. The difference in baseline data was not statistically significant. The operation time of the deep neuromuscular block group was longer than moderate neuromuscular block group (*P* = 0.008), the dosage of propofol and remifentanil was less than moderate neuromuscular block group (*P* = 0.043; *P* < 0.001), the dosage of rocuronium was higher than moderate neuromuscular block group (*P* < 0.001) and the length of hospital stay was shorter than moderate neuromuscular block group (*P* = 0.028). The differences were statistically significant.

**Table 1:** Baseline data and intraoperative characteristics

	Deep neuromuscular block group (n = 35)	Moderate neuromuscular block group (n = 35)	*P-*value
Age (years)	73.5 ± 6.4	71.6 ± 5.7	0.185
Sex (n)			0.803
Male	23 (65.7)	22 (62.9)	
Female	12 (34.3)	13 (37.1)	
BMI (kg m^−2^)	24.8 (3.2)	23.8 (2.6)	0.148
ASA physical status (n)			0.124
2	19 (54.3)	23 (65.7)	
3	16 (45.7)	12 (34.3)	
Operation time (min)	356.7 (107.6)	294.8 (80.0)	0.008
Anesthesia time (min)	430.3 (117.4)	384.3 (88.8)	0.069
Pneumoperitoneum time (min)	177.7 (69.5)	165.7 (49.8)	0.409
Pneumoperitoneum pressure adjustment (n)	8 (22.9)	2 (5.7)	0.088
L-SRS			0.552
4	8 (22.9)	7 (20.0)	
5	27 (77.1)	28 (80.0)	
Extubation time (min)	12.9 (8.5)	13.7 (6.1)	0.631
Propofol (mg·kg^−1^·h^−1^)	3.4 (0.7)	3.8 (1.0)	0.043
Remifentanil (μg·kg^−1^·min^−1^)	0.2 (0.06)	0.3 (0.07)	0.000
Rocuronium (mg)	158.3 (71.6)	66.9 (25.9)	0.000
Norepinephrine (μg·kg^−1^·min^−1^)	0.09 (0.07)	0.07 (0.04)	0.175
Length of stay (days)	18.4 (4.9)	22.0 (8.3)	0.028

Data are presented as mean (standard deviation) or absolute number (%)

*ASA* American Society of Anesthesiologists, *L-SRS* Leiden-surgical rating scale. Age, BMI, operation time, anesthesia time, pneumoperitoneum time, pneumoperitoneum pressure and extubation time were analyzed using two independent samples t-test, so as the dosage of propofol, remifentanil, rocuronium, norepinephrine and the length of stay. Sex, ASA and L-SRS were analyzed with Chi-square test

Table 2 shows the details of the postoperative follow-up of the two groups. MINS was not observed in both groups; 3 patients in the moderate neuromuscular block group showed increased cTnI after surgery but did not meet the MINS diagnostic criteria. The NRS score in the deep neuromuscular block group was lower 72 h after surgery, and the difference was statistically significant (*P* = 0.018). There was no significant difference in the overall incidence of postoperative cardiovascular complications between the two groups, among which the highest incidence was deep venous thrombosis (11.4%).

**Table 2:** Postoperative status and cardiovascular complications

	Deep neuromuscular block group (n = 35)	Moderate neuromuscular block group (n = 35)	*P*-value
MINS (n)	0 (0.0)	0 (0.0)	-
Postoperative pain (NRS)			
24 h	2 (2,3.75)	2 (3,4)	0.430
48 h	1 (2,2)	1 (2,3)	0.232
72 h	0 (1,2)	0 (1,2)	0.018
PONV (n)	10 (28.6)	16 (45.7)	0.138
Cardiovascular	6 (17.1)	9 (25.7)	0.382
complications (n)			
DVT (n)	5 (14.3)	3 (8.6)	0.770
Arrhythmia (n)	1 (2.9)	2 (5.7)	1.000
Hypotension (n)	0 (0.0)	3 (8.6)	0.239
Cardiac failure (n)	0 (0.0)	1 (2.9)	1.000

Data are presented as median (quartile) or absolute number (%)

*MINS* Myocardial injury after non-cardiac surgery, *NRS* Numeric rating scale, *PONV* Postoperative nausea and vomiting, *DVT* Deep venous thrombosis. Incidence of MINS, PONV and the number of cases of cardiovascular complications were analyzed with Chi-square test and the NRS was compared with Man-Whitney U test

**Fig. 2:**
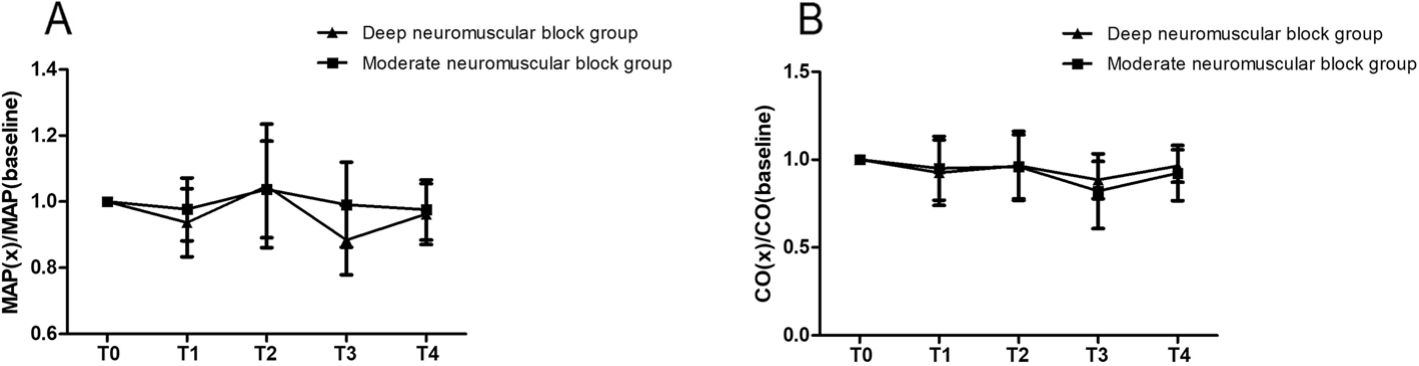
Ratio of intraoperative MAP and CO to baseline data. Figure 2A is the ratio of intraoperative MAP to MAP (baseline); Fig. 2B is the ratio of intraoperative CO to CO (baseline). T0, patients enter the operation room; T1, after establishing artificial pneumoperitoneum; T2, after the placement of operation position; T3, after stopping pneumoperitoneum; T4, before patients leaving the operation room. MAP: mean arterial pressure; CO: cardiac output

Figure 2 shows the intraoperative MAP and CO ratio to baseline data (T0). After stopping artificial pneumoperitoneum (T3), the ratio of MAP to baseline MAP in the moderate neuromuscular block group was higher than deep neuromuscular block group, and the difference was statistically significant (0.88 ± 0.11 vs 0.99 ± 0.13, *P* < 0.001). At other time points, the data differences between the two groups were small and with no statistical significance.

## Discussion

This study shows that in older patients who underwent laparoscopic colorectal cancer resection, there was no statistical difference in the MINS incidence and other cardiovascular adverse events within 7 days after surgery in the maintenance of deep neuromuscular block compared with moderate neuromuscular block.

Previous clinical studies indicated that preoperative, intraoperative and postoperative risk factors are correlated with MINS [17]. Preoperative risks were related to the age, gender, and previous medical history of the patients. Intraoperative risks were mainly related to sympathetic excitation, inflammation, blood loss, hypotension and secondary arrhythmia caused by anesthesia. The postoperative risks were related to the imbalance of myocardial oxygen supply and demand and coronary embolism caused by the above factors. In this study, all were older patients with complex preoperative complications and reduced compensatory function of the cardiovascular system. Based on the above factors, MINS incidence should be increased following earlier studies. Most MINS do not have typical clinical symptoms of myocardial ischemia. At present, the early diagnosis and screening of MINS mainly depend on the elevation of biomarkers. The guidelines issued by ESC/ESA suggest cTn detection before and 48–72 h after non-cardiac surgery in high-risk patients to identify the occurrence of myocardial injury, and monitoring of NT-proBNP may be considered to predict perioperative and long-term cardiovascular events in patients [18]. MINS was diagnosed by an elevation in cTn-levels exceeding the 99th percentile within the first 30 days after surgery [19]. cTnI and cTnT are routinely used to detect myocardial injury to their high specificity. In addition, elevated NT-proBNP has also been listed as a strong predictor for cardiovascular complications and mortality after noncardiac surgery [20]. CK-MB and Myo have not been used as independently predictors of MINS due to their high sensitivity but low specificity. Based on the above conclusions, this study used cTnI as an indicator and dynamically observed the changes within 7 days after operation to predict MINS.

In addition, according to ACC/AHA guidelines of 2014, intraperitoneal surgery is a medium-risk surgery with a 1–5% risk of cardiovascular death and myocardial infarction within 30 days after surgery [21]. With the increase of artificial pneumoperitoneum pressure in laparoscopic surgery, the cardiac afterload is increased and the preload and cardiac output is decreased gradually, but circulation collapse associated with moderate neuromuscular block has not been observed. Cho et al. compared the average values of cardiac index 30 min postoperative of standard pneumoperitoneum pressure + moderate neuromuscular block, low pneumoperitoneum pressure + deep neuromuscular block and standard pneumoperitoneum pressure + deep neuromuscular block during laparoscopic rectal surgery, and no statistical difference was observed [22]. In addition, increased abdominal pressure led to significant increases in tumor necrosis factor α, interleukin-1β, interleukin-6, interleukin-8, and C-reactive protein after surgery, and maintaining deep neuromuscular block during surgery does not reduce the stress response of patients [23]. Moderate neuromuscular block and high pneumoperitoneum pressure affect patients' microcirculation. Liu et al. verified that the blood flow of superior mesenteric artery and hepatic portal vein will decrease if pneumoperitoneum pressure is higher than 14 mmHg [24]. The organs will suffer from ischemia under insufficient microcirculation perfusion, while oxygenated blood reperfusion at the end of pneumoperitoneum enhances the body oxidation reaction, and oxygen free radicals generated during myocardial ischemia–reperfusion promote the apoptosis of cardiomyocytes [25]. Intestinal ischemia–reperfusion reaction is also one of the potential mechanisms of oxidative stress injury of cardiomyocytes and cardiac vessels [26]. Based on the above evidence, this study assumed that different degrees of neuromuscular block associated with the incidence of MINS after surgery by influencing internal abdominal pressure.However, this study failed to obtain results consistent with earlier reports and common assumptions. This could be because, at first, this study used propofol, sufentanil and rocuronium for sequential induction. As a powerful opioid analgesic, sufentanil inhibits adrenaline secretion and increases vagal tone, thus avoiding large fluctuations in blood pressure and heart rate during the operation [27]. This study used 0.3 µg/kg sufentanil for anesthesia induction. An earlier study demonstrated that this sufentanil dose could effectively inhibit the stress response during anesthesia induction and endotracheal intubation [28]. The stimulation during endotracheal intubation is one of the main reasons for the circulation fluctuation during anesthesia induction. In this study, the appropriate dose of sufentanil was administered to minimize the additional cardiac work and avoid the significant increase in myocardial oxygen consumption caused by endotracheal intubation, thus reducing myocardial ischemia incidence.

Secondly, establishing an artificial pneumoperitoneum leads to increased intraperitoneal pressure, intraperitoneal venous return obstruction and hypotension secondary to cardiac preload insufficiency. In this study, MAP and CO in both groups decreased after establishing pneumoperitoneum than the baseline level. However, laparoscopic colorectal resection is performed under the Trendelenburg position. Previous studies have shown that the 45° Trendelenburg position increases venous return and mean arterial pressure during laparoscopic prostatectomy more than the supine position [29]. At the same time, CO and heart rate do not change significantly. This is consistent with the results observed in this study. MAP increased by 10% in the deep neuromuscular block group and 6% in the moderate neuromuscular block group after position placement than earlier, compensating for the inhibitory effect of increased intraperitoneal pressure on blood return to some extent.

In addition, low doses of norepinephrine were used continuously during surgery to maintain appropriate peripheral vascular tone, and MAP was maintained within ± 20% of baseline levels. Previous studies have suggested that intraoperative MAP < 65 mmHg, or about 20% lower than the baseline value, is associated with myocardial injury [30]. Esther et al. analyzed the correlation between intraoperative hypotension exposure and adverse patient outcomes, and the results showed a hierarchical relationship between intraoperative hypotension and postoperative myocardial injury and mortality. The severity of hypotension seemed more important than the duration [31], and individual maintenance based on the preoperative baseline blood pressure level was more significant than the simple control of hypotension. The previous study also exhibited that the ability of intraoperative use of norepinephrine to control average systolic blood pressure above the preset target was significantly higher than the placebo group and increased cardiac output through the β-receptor excitation of norepinephrine, especially in the lower concentration range [32]. Combining the above two may further offset the changes in MAP and CO caused by different degrees of neuromuscular block.

Thirdly, postoperative pain is significantly associated with MINS. Turan et al. observed 2892 patients who underwent non-cardiac surgery, among which 4.5% of patients experienced MINS within 72 h after surgery [33]. The time-weighted average (TWA) pain score of patients with MINS was 4.5 (SD = 2.0), and the TWA pain score and MINS predicted value illustrated an approximately linear increase in monotone. This indicates that the higher the postoperative TWA pain score, the higher the risk of MINS. Active and effective pain management during the perioperative period may significantly improve the prognosis of patients. Patients in the two groups received postoperative multimodal analgesia through incision local anesthetic infiltration and patient-controlled intravenous analgesia. The median pain NRS score within 72 h after surgery was ≤ 2, and the difference was not statistically significant. Therefore, good postoperative pain management may be one of the reasons for the absence of MINS in both groups.

Moreover, active body temperature management might also be one of the reasons for the low MINS incidence. Older patients are more prone to hypothermia after general anesthesia due to their low metabolic rate and poor thermoregulation function. Previous studies indicated that for older patients undergoing radical esophagectomy, intraoperative temperature management, active physical heating, and thermal insulation could effectively reduce MINS incidence and severe arrhythmia within 2 days after surgery [34]. In this study, all subjects were actively insulated. The core temperature during the operation was maintained at least 36 ℃ by infusing a heated liquid and using an electric blanket and heater. This integrated management may benefit older patients by reducing intraoperative and postoperative cardiovascular adverse events due to low or rapid core temperature reduction.

Finally, sugammadex sodium was routinely used for muscle relaxation antagonism. In older patients, organ function declines, and respiratory muscle strength decreases, leading to residual muscle relaxation and early postoperative low oxygen saturation events after surgery. A previous study showed that 57.7% of older patients (70–90 years old) suffered from residual muscle relaxation postoperatively, and the duration of PACU stay and hospital stay were significantly longer than younger patients [35]. Sugammadex sodium is a rocuronium-specific antagonist that reverses deep and moderate neuromuscular blocks at 4 mg/kg and 2 mg/kg, respectively, with significantly reduced risk of residual muscle relaxations and fewer respiratory and cardiovascular events than neostigmine [36, 37]. Sugammadex sodium was given before anesthesia recovery, and the duration of circulation fluctuation and imbalance of myocardial oxygen supply and demand were shortened from awakening to extubation. This might also be why the MINS incidence in this study was lower than in previous studies, and there was no significant difference between the two groups.

This study has some limitations. First, MINS is defined as a myocardial injury occurring within 30 days after surgery. Due to the limited hospital stay of patients, it is difficult to perform a myocardial marker examination 30 days after surgery. A previous study has reported that 94% of MINS occur within 48 h after surgery [5]. Although our follow-up time has covered most of the MINS occurrence time, there could be some omissions. Secondly, studies have shown that the sensitivity of traditional cTn analysis is low in the low concentration range and may not provide reliable information in the perioperative period [38]; hs-cTn may be a more sensitive biological marker. VISION2017 believes that hs-cTnT elevation should be the diagnostic criteria for MINS [5], and hs-cTnT detection can be increased to improve the detection rate of MINS further. Finally, MINS incidence was lower than reported in the earlier study, possibly due to the followed anesthesia management strategy in this study or the small sample size. Future studies can expand the sample size and grade patients according to their age and preoperative complications for exploration.

## Conclusion

This study confirmed that deep neuromuscular block could reduce the intraoperative dosage of propofol and remifentanil, shorten the length of hospital stay and reduce the patients’ pain intensity 72 h after surgery, which were partially consistent with the previous studies. Maintaining different degrees of the neuromuscular block under TOF guidance intraoperatively did not change the MINS incidence within 7 days after surgery. Therefore, concern about the occurrence of MINS cannot be taken as a reason to refuse the application of moderate neuromuscular block in older patients undergoing laparoscopic colorectal cancer resection.

## Data Availability

The datasets used and/or analyzed during the current study are available from the corresponding author on reasonable request.

## Abbreviations

MINS: Myocardial injury after non-cardiac surgery
cTnI: Cardiac troponin I
cTnT: Cardiac troponin T
ERAS: Enhanced recovery after surgery
CO: Cardiac output
ECG: Electrocardiogram
BIS: Bispectral index
MAP: Mean artery pressure
TOF: Train of four
PTC: Post-tetanic count
PCV-VG: Pressure-controlled volume guaranteed ventilation
PEEP: Positive end-expiratory pressure
TIVA: Total intravenous anesthesia
L-SRS: Leiden-surgical rating scale
NRS: Numerical rating scale
TWA: Time-weighted average
